# Brusatol Inhibits Proliferation and Invasion of Glioblastoma by Down-Regulating the Expression of ECM1

**DOI:** 10.3389/fphar.2021.775680

**Published:** 2021-12-14

**Authors:** Zhang’an Dai, Lin Cai, Yingyu Chen, Silu Wang, Qian Zhang, Chengde Wang, Ming Tu, Zhangzhang Zhu, Qun Li, Xianghe Lu

**Affiliations:** ^1^ Department of Neurosurgery, The First Affiliated Hospital of Wenzhou Medical University, Wenzhou, China; ^2^ Department of Neurosurgery, Shanghai Jiao Tong University Affiliated Sixth People’s Hospital, Shanghai, China

**Keywords:** brusatol, glioblastoma, proliferation, invasion, extracellular matrix protein 1

## Abstract

Brusatol (Bru), a Chinese herbal extract, has a variety of anti-tumor effects. However, little is known regarding its role and underlying mechanism in glioblastoma cells. Here, we found that Bru could inhibit the proliferation of glioblastoma cells *in vivo* and *in vitro*. Besides, it also had an inhibitory effect on human primary glioblastoma cells. RNA-seq analysis indicated that Bru possibly achieved these effects through inhibiting the expression of extracellular matrix protein 1 (ECM1). Down-regulating the expression of ECM1 *via* transfecting siRNA could weaken the proliferation and invasion of glioblastoma cells and promote the inhibitory effect of Bru treatment. Lentivirus-mediated overexpression of ECM1 could effectively reverse this weakening effect. Our findings indicated that Bru could inhibit the proliferation and invasion of glioblastoma cells by suppressing the expression of ECM1, and Bru might be a novel effective anticancer drug for glioblastoma cells.

## Introduction

Glioblastoma multiforme (GBM) is the most common primary intracranial malignant tumor, accounting for 48–60% of intracranial tumors in clinical practice (Keles and Berger, 2004; Wen and Kesari, 2008). Even though certain progress has been made in the study of GBM, the life expectancy of patients with GBM has not been significantly improved (Gilbert et al., 2013; Kaur et al., 2020). Temozolomide (TMZ), the first choice for GBM treatment, only slightly improves the median overall survival (MOS) of these patients. In comparison with patients treated with postoperative radiotherapy alone, the MOS of patients treated with postoperative radiotherapy combined with TMZ was only prolonged by 5 months (McAleenan et al., 2021). On the cellular level, the effectiveness of TMZ is significantly limited by the methylation of O^6^-methylguanine DNA methyltransferase (MGMT) gene promoter. TMZ triggers cytotoxicity increase and cell apoptosis of GBM tumor cells by alkylating the O^6^-position of guanine, while MGMT is the protein that allows DNA repair by removing the alkyl group from the O^6^-position of guanine and eventually results in tumor resistance to alkylating agents. MGMT promoter methylation can cause MGMT expression deficiency, thereby promoting tumor cell sensitivity to alkylating drugs, such as TMZ (Hegi et al., 2005). Hence, not all GBM patients can benefit from TMZ treatment (Hegi et al., 2019), especially for those patients without MGMT promoter methylation. In addition, even though TMZ had an anti-GBM effect (Hegi et al., 2019), the MOS of patients with MGMT promoter hypermethylated was only 18.4 months, compared to 6.7 months for those patients without MGMT promoter methylation (Wick et al., 2020). Therefore, there is an urgent need for the development of new therapeutic drugs and the identification of novel drug targets for GBM treatment.

Brusatol (Bru, known as Ya-Dan-Zi in Chinese), a compound first isolated and characterized from the seeds of *Brucea sumatrana* in 1968, has been traditionally used for the treatment of dysenteric disorders, malaria, and amebiasis (Cai et al., 2019). With the rapid development of medical chemistry, Bru has been recognized as an effective anticancer agent in multiple types of tumor cells (Cai et al., 2019). Hall et al. (Hall et al., 1979) found that Bru exhibited potent suppression effect on tumor cell metabolism and proliferation of lymphocytic leukemia cells by remarkably inhibiting the synthesis of c-myc. Subsequently, Olayanju et al. (Olayanju et al., 2015) proved that Bru could cause rapid and transient depletion of Nrf2 protein through a post-transcriptional mechanism, which significantly inhibited the proliferation of liver cancer cells. Several pioneering findings also showed that Bru was effective in the treatment of digestive system tumors (Lu et al., 2017; Liu et al., 2018; Ye et al., 2018), respiratory system tumors (Ren et al., 2011; Sun et al., 2016), blood system tumors (Hall et al., 1979; Hall et al., 1981; Mata-Greenwood et al., 2002), and melanoma (Wang et al., 2018). Recent findings suggested that Bru inhibited glioma proliferation *in vitro* and *in vivo* by down-regulating the expression of Nrf2 (Tang et al., 2020). Although the underlying mechanism of Bru in GBM has been initially explored, the detailed pharmacological effects of Bru cannot be fully explained by the studies conducted only on one target of action, and the mechanism needs to be further validated on human primary GBM cells. Therefore, we hope to further explore the role of Bru in GBM treatment through more in-depth studies.

In the present study, we aimed to investigate the anticancer effects and the underlying mechanism of Bru in glioblastoma cells, and we found that Bru could induce the cell apoptosis of glioblastoma cells *in vitro* and *in vivo via* down-regulating the expression of ECM1. Regulating the expression of ECM1 could affect the efficacy of Bru treatment. Moreover, Bru could also exert its inhibitory effect on human primary GBM cells. These results suggest that Bru is a promising chemotherapeutic drug that is highly effective against glioblastoma cells.

## Materials and Methods

### Cell Culture and Reagents

The human GBM cell lines A172, U251, U87, used in this study, were purchased from the Cell Bank of the Chinese Academy of Sciences (Shanghai China). All cell lines were cultured in Dulbecco’s modified eagle medium (DMEM, Gibco, United States, #8121300), supplemented with 10% fetal bovine serum (FBS, Gibco, United States, #10099-141) and 100U/mL penicillin/streptomycin (Gibco, United States, #15140-122) and were incubated in a humidified atmosphere with 5% (v/v) CO_2_ at 37°C. Bru was purchased from MCE (Medchemexpress, United States, #HY-19543) and dissolved in DMSO.

### GBM Tumor Tissue Collection

This study was approved by the Clinical Research Ethics Committee of the First Affiliated Hospital of Wenzhou Medical University (permission: 2015-063), and written informed consents were obtained from all patients. The study protocol was carried out according to the principles of the Helsinki Declaration. Ten GBM patients (Grade IV), who had no history of preoperative radiotherapy or chemotherapy and underwent surgery between 2019 and 2021 at the First Affiliated Hospital of Wenzhou Medical University, were included in this retrospective research to be analyzed for ECM1 expression. The samples were taken from nine males and one female, aged between 48 and 77 years (mean 66.7 years). The clinical characteristics of the patients are shown in Table 1.

**TABLE 1:** Clinicopathological characteristics of 10 patients with glioblastoma.

Patient number	Gender	Age	Treatment method	Treatment after surgery	Diagnosis time	Last treatment time	Max diameter (cm)	ECM1 IHC	MGMT	IDH1
1	M	77	Sur	Un^a^	2020.10.14	2021.02.24	5	−	+	-
2	M	68	Sur	T + R	2019.11.05	2020.09.23	6.5	−	−	−
3	M	76	Sur	T + R	2021.02.02	2021.04.09	6	+	+	−
4	M	67	Sur	T + R	2020.12.25	2021.09.05	1.2	+	−	−
5	M	48	Sur	T + R	2020.12.03	2021.09.06	7.5	−	+	−
6	M	64	Sur	T + R	2018.11.10	2020.01.22	3	−	−	−
7	M	69	Sur	T + R	2021.03.23	2021.08.30	5.2	+	+	−
8	M	70	Sur	T + R	2021.03.04	2021.08.12	5.5	+	−	−
9	M	63	Sur	T + R	2019.02.01	2021.09.06	5	+	+	+
10	F	65	Sur	T + R	2021.03.01	2021.08.09	5.7	+	−	−

F, female; M, male; MGMT, O-6-methylguanine-DNA methyltransferase; IDH1, isocitrate dehydrogenase 1 Sur, Surgery; Un, untreatment; T, temozolomide; R, radiotherapy.

a The patients were intolerant to chemoradiotherapy.

For the isolation of human primary GBM cells, all steps were carried out under sterile conditions in a laminar flow cell culture hood to reduce the risk of contamination of the primary culture. Firstly, the tumor specimens were washed with phosphate buffered saline (PBS, Gibco, United States, #70011069) and dissected into small fragments. Then, the fragments were enzymatically digested by using human Tumor Dissociation kit (Miltenyi Biotec, Germany, #130-095-929) in combination with the gentleMACS™ Dissociator (Miltenyi Biotec, Germany, #DXT-130-096-730). Fibroblasts containing in the cell suspension were removed by using human Anti-Fibroblast MicroBeads (Miltenyi Biotec, Germany, #130-050-601) and erythrocytes were removed by using Red Blood Cell Lysis Solution (10×) (Miltenyi Biotec, Germany, #130-094-183). Finally, a total of 1 × 10^6^ cells were seeded in T25 cell culture flasks with a medium composed of DMEM medium (Gibco, United States, #8121300), 10% FBS (Gibco, United States, #10099-141) and 100U/mL penicillin/streptomycin (Gibco, United States, #15140-122) at 5% (v/v) CO2 humidified atmosphere with 37°C for further research.

### Cell Viability and Colony Formation Assay

Cell Counting Kit-8 (CCK-8; Dojindo, Japan, #CK04) was used to analyze cell viability. Three GBM cell lines (A172, U251 and U87) were plated in 96-well plates with a density of 10^4^ cells/well and incubated for 24 h as per the manufacturer’s instructions. At the indicated times after treatment, 10 μL of CCK-8 solution was added to 90 μL of culture medium. The cells were subsequently incubated for 3 h at 37°C in 5% (v/v) CO_2_ and the optical density was measured at 450 nm using a plate reader (TECAN, Switzerland). In colony formation assay, the cells were plated into six-well plates with a density of 500 cells/well and cultured for 2 weeks. Then, different concentrations of Bru or DMSO alone as control were added to treat the cells for 24 h. Next, cells were stained with the Crystal Violet Staining Solution (Beyotime, China, #C0121) according to the manufacturer’s instructions. Furthermore, microscopic examination was performed using an Axiovert 200 microscope (Carl Zeiss, Oberkochen, Germany), and cell colonies were counted using PhotoShop CS6. Three independent experiments were performed.

### Tumor Formation Assay

Six-week-old female BALB/c (nu/nu) athymic nude mice were purchased from Shanghai Experimental Animal Center (Shanghai, China). U87 cells (1×10^7^) were resuspended in PBS with Matrigel (1:1; BD Biosciences, United States, #356234) and then subcutaneously injected into the right back of each nude mouse. All mice had free access to food and water. When the transplanted tumors reached an average size of 50 mm^3^, the mice were randomly assigned to two groups. Vehicle or Bru was administered (2 mg/kg) daily by oral gavage. Tumor volumes were measured with calipers at two perpendicular diameters and calculated individually using the formula (length×width^2^)/2. Twenty-five days later, all mice were sacrificed, and the tumors were harvested. All procedures were approved by the Administration Committee of Experimental Animals, Laboratory Animal Center, Wenzhou Medical University (Permit Number: wydw 2020-146).

### Cell Death Analysis by Flow Cytometry

To determine the effect of Bru on cell apoptosis, a FITC Annexin V apoptosis detection kit I (BD Biosciences, United States, #559763) was used according to manufacturer’s instructions. First, the cells were treated with Bru for 0–72 h and collected. Then, a total of ≥10,000 cells were resuspended in binding buffer and incubated with 5 µL of Annexin V-FITC and 5 µL of PI for 15 min in dark. The proportions of cells in early apoptosis and late apoptosis were reported as the percentage of Annexin V^+^/PI^−^- and Annexin V^+^/PI^+^-labeled cells, respectively. The stained cells were analyzed directly by flow cytometry using a FACSCalibur with the Cell Quest program (BD Biosciences, United States) for data analysis.

### Western Blot Analysis

Cell lysates and tumor samples were extracted with cell lysis buffer (Beyotime, China, #P0013J), and the protein concentrations in the lysates were quantified using an Enhanced BCA Protein Assay Kit (Beyotime, China, #P0010). Protein samples (30–50 µg) were separated by SDS-PAGE and then transferred to PVDF membranes (Millipore, United States, #IPVH00010). The membranes were immunoblotted with primary antibodies followed by HRP-conjugated secondary antibodies. Immunoreactive proteins were visualized using an ECL Western blot detection kit (Advansta, United States, #K12045), and images were developed using a Bio-Rad system (Bio-Rad, United States). The indicated antibodies are listed as follow: rabbit anti-cleaved-Caspase-3 antibody (molecular weight: 17 kDa, 1:1,000; Cell Signaling Technology; #9664T), rabbit anti-pro-Caspase-3 antibody (molecular weight: 35 kDa, 1:1,000; Cell Signaling Technology; #14220T), rabbit anti-cleaved-Caspase-9 antibody (molecular weight: 37 kDa, 1:1,000; Cell Signaling Technology; #52873T), mouse anti-pro-Caspase-9 antibody (molecular weight: 47 kDa, 1:1,000; Cell Signaling Technology; #9508T), rabbit anti-Bax antibody (molecular weight: 21 kDa, 1:1,000; Affinity; #AF0120), rabbit anti-Bcl-2 antibody (molecular weight: 26 kDa, 1:1,000; Affinity; #AF6139), rabbit anti-ECM1 antibody (molecular weight: 61 kDa, 1:1,000; Abcam; #ab126629), rabbit anti-MMP1 antibody (molecular weight: 54 kDa, 1:1,000; Affinity; #AF0209), rabbit anti-TIMP1 antibody (molecular weight: 24 kDa, 1:1,000; Affinity; #AF7007), rabbit anti-MMP2 antibody (molecular weight: 72 kDa, 1:1,000; Abcam; #ab181286), rabbit anti-TIMP2 antibody (molecular weight: 24 kDa, 1:1,000; Affinity; #AF0264), rabbit anti-MMP9 antibody (molecular weight: 78 kDa, 1:1,000; Abcam; #ab76003), mouse anti-beta-Actin antibody (molecular weight: 42 kDa, 1:1,000; Affinity; #T0022), rabbit anti-Tubulin beta antibody (molecular weight: 55 kDa, 1:1,000; Affinity; #AF7011). Three independent experiments were performed.

### RNA-Sequence Array

In total, 3 µg of RNA per sample was used as input material for the preparations. Sequencing libraries were generated using the NEB Next® UltraTM RNA Library Prep Kit for Illumina® (NEB, United States, #E7530L) following the manufacturer’s recommendations, and index codes were added to attribute sequences to each sample. Clustering of the index-coded samples was performed on a cBot Cluster Generation System using a TruSeq PE Cluster Kit v3-cBot-HS (Illumina, United States, #PE-401-3001). After cluster generation, the library preparations were sequenced on an Illumina platform, and 125bp/150bp paired-end reads were generated.

### Real-Time PCR

Total RNA was extracted from GBM cells by using TRIzol™ reagent, according to the manufacturer’s instructions. The RNA concentrations were examined by a spectrophotometer (NanoDrop 1000, Thermo). The first-strand cDNAs were synthesized using a cDNA Synthesis SuperMix kit (YEASEN, China, #11123ES10). Each cDNA (2 μL) was amplified using the Hieff qPCR SYBR Green Master Mix (final volume, 20 ml, YEASEN, China, #11203ES03) and then analysed on the Applied Biosystems 7900 Real-time PCR Detection System. Thermal cycling conditions were performed as follows: melting step (95°C for 30 s), annealing step (40 cycles of 95°C for 10 s), and elongation (60°C for 30 s). Actin was used as internal control. Melting curves were used for verifying the specificity of each PCR reaction. The cycle threshold (Ct) was the mean value of three Ct values. The relative expression level of RNA was analyzed by the 2^−ΔΔCt^ method.

RNA primers are listed as follows:

ECM1 (human-forward): 5′-TGA​ACC​AAA​TCT​GCC​TTC​CTA​AC-3’
ECM1 (human-reverse): 5′-GCT​GGA​CTG​TGG​TAG​GTT​CCA-3’
GAPDH (human-forward): 5′-CGA​AAT​CCC​ATC​ACC​ATC​TTC​CAG​G-3’
GAPDH (human-reverse): 5′-GAG​CCC​CAG​CCT​TCT​CCA​TG-3’.

### Gene Silencing and Overexpression

Three GBM cell lines (A172, U251 and U87) were transfected with small interfering (si)RNA oligonucleotides by riboFECT^TM^ CP Transfection kit (RIBOBIO, China, #C10511). Briefly, siRNA was first diluted with riboFECT^TM^ CP Buffer and then co-incubated with riboFECT^TM^ CP Reagent for 15min at room temperature. The mixture was then applied to cells plated in 2 ml of medium (final concentration of siRNA, 50 nM). All siRNAs were purchased from RIBOBIO.

The sequences of siRNAs were as follows:

siECM1: 5′-GCT​TCA​ACA​TCA​ATT​ATC​T-3'
siControl: 5′-GGC​TCT​AGA​AAA​GCC​TAT​GC-3'

Nucleotide sequence coding for ECM1 was cloned into pLV-CMV-ECM1-EF1-ZsGreen1-T2A-Puro (Youze Bio, China, #LV-0368) packaged by lentivirus. According to the manufacturer’s instructions, the three GBM cell lines (A172, U251 and U87) were infected with lentivirus expressing ECM1 (ECM1) or empty lentivirus (Control) for 96 h and then used separately for further studies. All lentiviruses were labeled with ZsGreen1.

### Wound-Healing Assay

Migration of 3 cell lines (A172, U251 and U87) was measured by determining the ability of cells to move into cell-free spaces. Cells were treated with siRNA or lentivirus as mentioned. When cells reached confluence, sterile plastic pipette tips were used to form identical linear wounds. At 0 and 48 h, the scratches were photographed and evaluated using an inverted phase contrast microscope. Migration distances were then calculated.

### Transwell Invasion Assay

Matrigel solution was prepared by diluting Matrigel with serum-free cell culture medium at a dilution of 1:8. The 24-well Transwell chambers (Corning Costar, United States, #3422) were coated with Matrigel solution for 12 h at 37°C before cell seeding. Cells were cultured in the chamber with serum-free medium containing 1% bovine serum albumin in triplicate at 10^5^ cells per well. After incubation at 37°C for 48 h, the chambers were gently wiped with a cotton swab. Cells invading the polycarbonate membrane were fixed with 4% paraformaldehyde for 15 min and then washed with PBS twice for a total of 10 min. Staining was done with 0.1% crystal violet for 10min and then washed twice with PBS. The cell numbers on the lower surface of the membrane were counted under Axiovert 200 microscope (Carl Zeiss, Oberkochen, Germany).

### Immunohistochemical Analysis

Formalin-fixed tumor tissues were fixed in 4% paraformaldehyde for 24 h, embedded in paraffin, and then serially sectioned (4 μm). For hematoxylin and eosin (H&E) staining, sections were stained with H&E (Beyotime, China, #P0010). For IHC, sections were stained with primary antibodies against ECM1 (Abcam, United States, #ab126629). Briefly, the tissue sections were dehydrated and subjected to peroxidase blocking. Primary antibody was added and incubated at room temperature for 30 min on the Dako AutoStainer using the Dako Cytomation EnVision + System-HRP (DAB) detection kit (Carpinteria, United States, #K406511). Slides were counterstained with hematoxylin and then observed under a microscope.

### Statistical Analysis

All statistical analyses were performed using the SPSS 17.0 statistical software (SPSS Inc., Chicago, IL, United States). Continuous variables were presented as mean ± SE. Statistical significance between two measurements was determined by the two tailed unpaired Student’s *t*-test, and among groups, it was determined by one-way analysis of variance (*ANOVA*) followed by Tukey’s post-hoc multiple comparison test. *p* values <0.05 were considered as statistically significant.

## Results

### Bru Inhibited Proliferation of GBM Cells *in Vivo* and *in Vitro*

**FIGURE 1:**
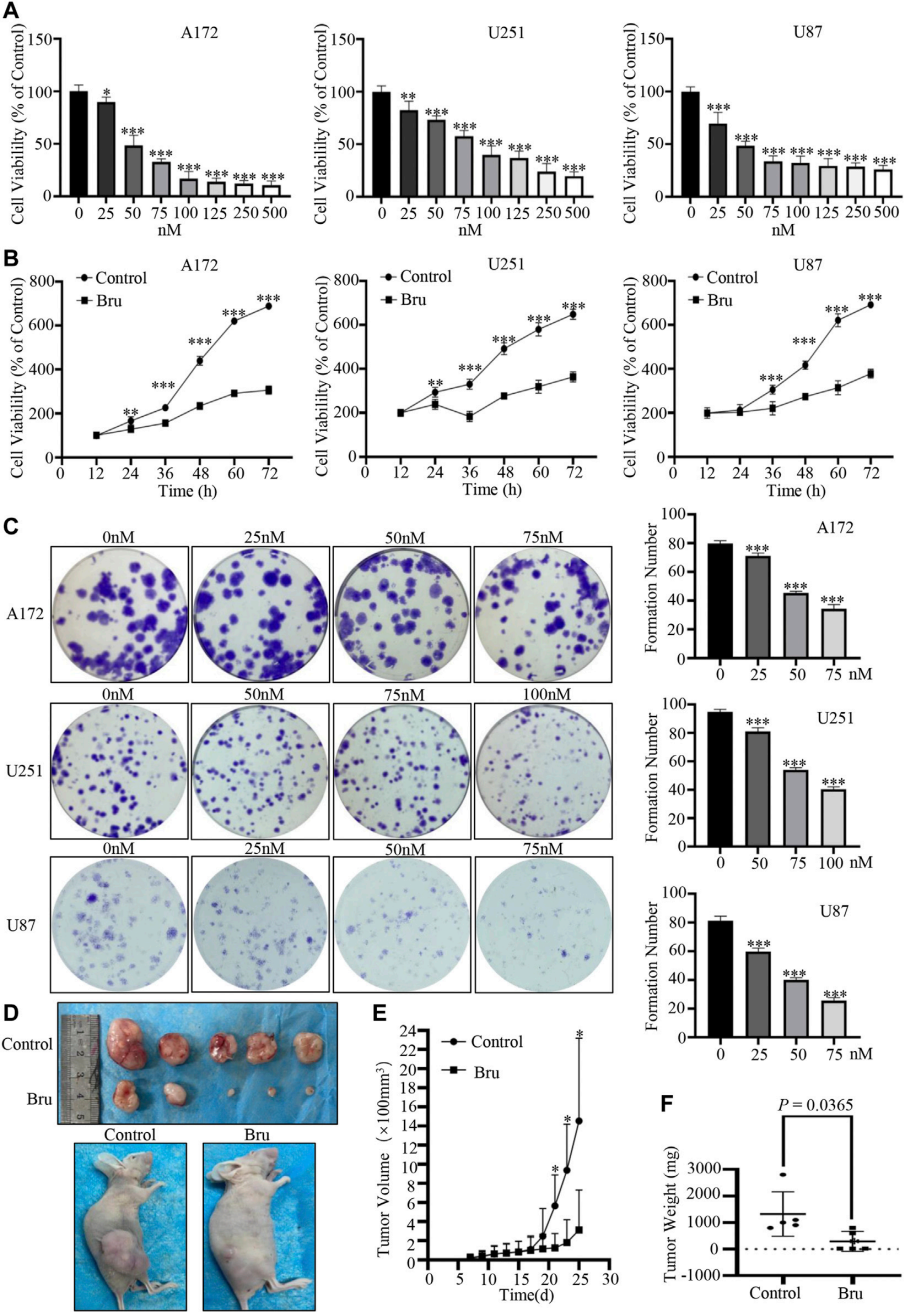
Bru inhibited GBM cells proliferation *in vivo* and *in vitro.* **(A)** The proliferation-suppressive effect of Bru was determined by cell viability assay. As Bru concentration increased, the viability of three GBM cell lines A172 cells **[** **(A)**, **left]**, U251 cells **[** **(A)**, **middle]**, and U87 cells **[** **(A)**, **right]** significantly decreased. **(B)** The inhibitory effect of Bru on three GBM cell lines increased significantly over time, which was determined by cell viability assay. A172 cells [**(B)**, left, 50 nM], U251 cells [**(B)**, middle, 75 nM], and U87 cells [**(B)**, right, 50 nM] were treated with Bru for 12–72 h and then subjected to cell viability assay. **(C)** The clone formation assay of three GBM cell lines. Bru treatment resulted in the inhibition of cell colonies. **(D)** Bru treatment (2 mg/kg, daily by oral gavage) inhibited the growth of subcutaneously transplanted U87 cell tumors in nude mice. Representative images of xenograft tumors from nude mice were shown in Panel **(D)**. **(E)** Assessment of the tumor growth curves in nude mice after Bru treatment. **(F)** The weight of xenograft tumors from nude mice were shown in Panel **(F)**. *p < 0.05, **p < 0.01, ***p < 0.001 vs, 0 nM group or Control group.

Firstly, cell viability and colony formation assay were used to clarify the cytotoxic effect of Bru on GBM cells. The results of cell viability assay showed that cell viability of three GBM cell lines (A172, U251, U87) significantly decreased in both dose- (Figure 1A) and time-dependent (Figure 1B) manner in response to Bru treatment. Similar results were also obtained from the plate clone formation assays in three GBM cell lines, which were treated with different concentrations of Bru for 24 h, determined by the formation count (Figure 1C). The formation number of A172 cells colonies was 79.8 ± 0.86 in 0 nM group, 71.2 ± 0.86 in 25 nM group, 45.4 ± 0.51 in 50 nM group, and 34.4 ± 1.29 in 75 nM group. The formation number of U251 cell colonies was 94.8 ± 0.80 in 0 nM group, 81 ± 1.18 in 50 nM group, 54 ± 0.71 in 75 nM group, and 40.4 ± 0.75 in 100 nM group, whereas the formation number of U87 cell colonies was 81.2 ± 1.43 in 0 nM group, 59.8 ± 1.07 in 25 nM group, 40 ± 0.71 in 50 nM group, and 25.6 ± 0.93 in 75 nM group. To further evaluate whether Bru-induced cell death was also effective *in vivo*, we investigated tumor growth in U87 xenograft models (Figure 1D). The results showed that the average tumor volume of Bru-treated group was significantly lower than that of Control group after transplanting for 25 days (3.13 ± 1.87 mm^3^ vs. 14.53 ± 3.87 mm^3^, n = 5, *p* = 0.0293; Figure 1E). The average tumor weight was 1,320 ± 373.36mg and 292 ± 169.54 mg in Bru-treated group and Control group, respectively (*p* = 0.0365, Figure 1F).

To conclude, these results suggested that Bru possessed a potent inhibitory effect on GBM cells *in vivo* and *in vitro*.

### Bru Induced Apoptosis in GBM Cells

**FIGURE 2:**
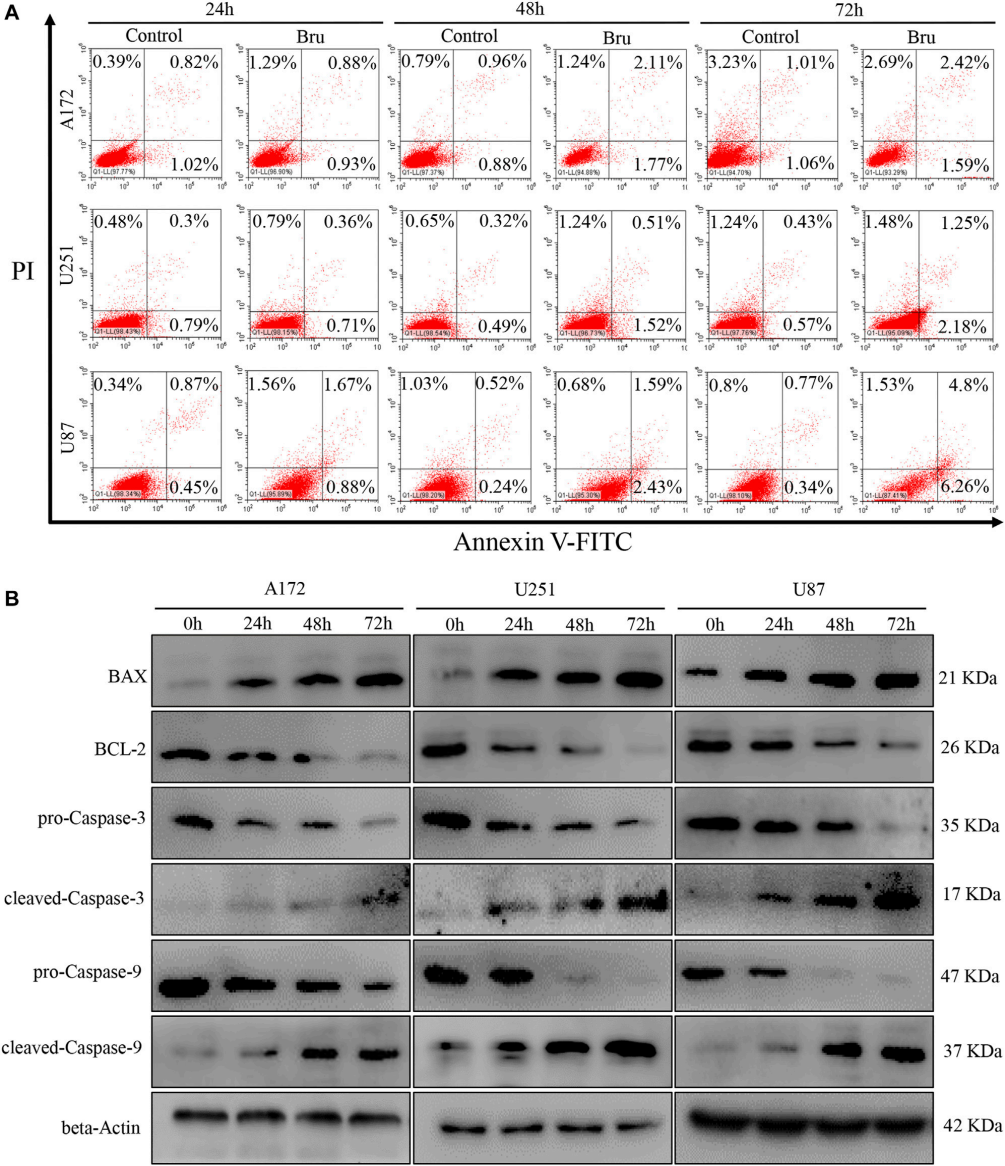
Bru induced apoptotic cell death of glioblastoma cells. **(A)** The cell apoptosis of A172 cells, U251 cells, and U87 increased obviously after treatment with Bru by flow cytometry analysis. The results revealed that Bru indeed rendered A172 cells, U251 cells, and U87 to undergo apoptosis. **(B)** A172, U251, and U87 cells were treated with Bru as indicated, and apoptosis-associated proteins were analyzed by Western blot using antibodies against Bax, Bcl-2, pro-Caspase-3, cleaved-Caspase-3, pro-Caspase-9, and cleaved-Caspase-9. Beta-Actin was used to control the consistency of protein loading.

Recently, mounting evidence has proved that Bru can inhibit the tumor growth *via* inducing cell apoptosis (Chen et al., 2018; Wang et al., 2018). In order to clarify whether this inhibitory effect of Bru on GBM cells was also related to the induction of apoptosis, we subjected Bru-treated cells to flow cytometry and western blot analyses. The results of Annexin V/PI apoptosis analysis demonstrated that Bru treatment increased the numbers of both early and late apoptotic cells in a time-dependent manner (Figure 2A). After being treated with Bru for 72 h, the percent of early apoptotic cells increased by 0.53% in A172 cells (*p* < 0.001), by 1.61% in U251 cells (*p* < 0.001), and by 5.92% in U87 cell (*p* < 0.001). Meanwhile, the percent of late apoptotic cells increased by 1.41% in A172 cells (*p* < 0.001), by 0.82% in U251 cells (*p* < 0.001), and by 4.03% in U87 cells (*p* < 0.001), compared with those in Control group (treated with DMSO alone). To further clarify the mechanism of Bru’s inhibitory effect on GBM cells, we investigated the expression of the main mitochondrion-dependent apoptotic proteins, including Caspase-3, Caspase-9, Bcl-2, and Bax in the three GBM cell lines by Western blotting analysis. As shown in Figure 2B, after Bru treatment, the levels of pro-Caspase-3, pro-Caspase-9, and Bcl-2 decreased, while the levels of cleaved-Caspase-3, cleaved-Caspase-9, and Bax significantly increased in a time-dependent manner.

To sum up, these results revealed that Bru could exert its inhibitory effect on GBM cells by inducing cellular apoptosis.

### Bru Suppressed GBM Cells Growth by Down-Regulating the Expression of ECM1

**FIGURE 3:**
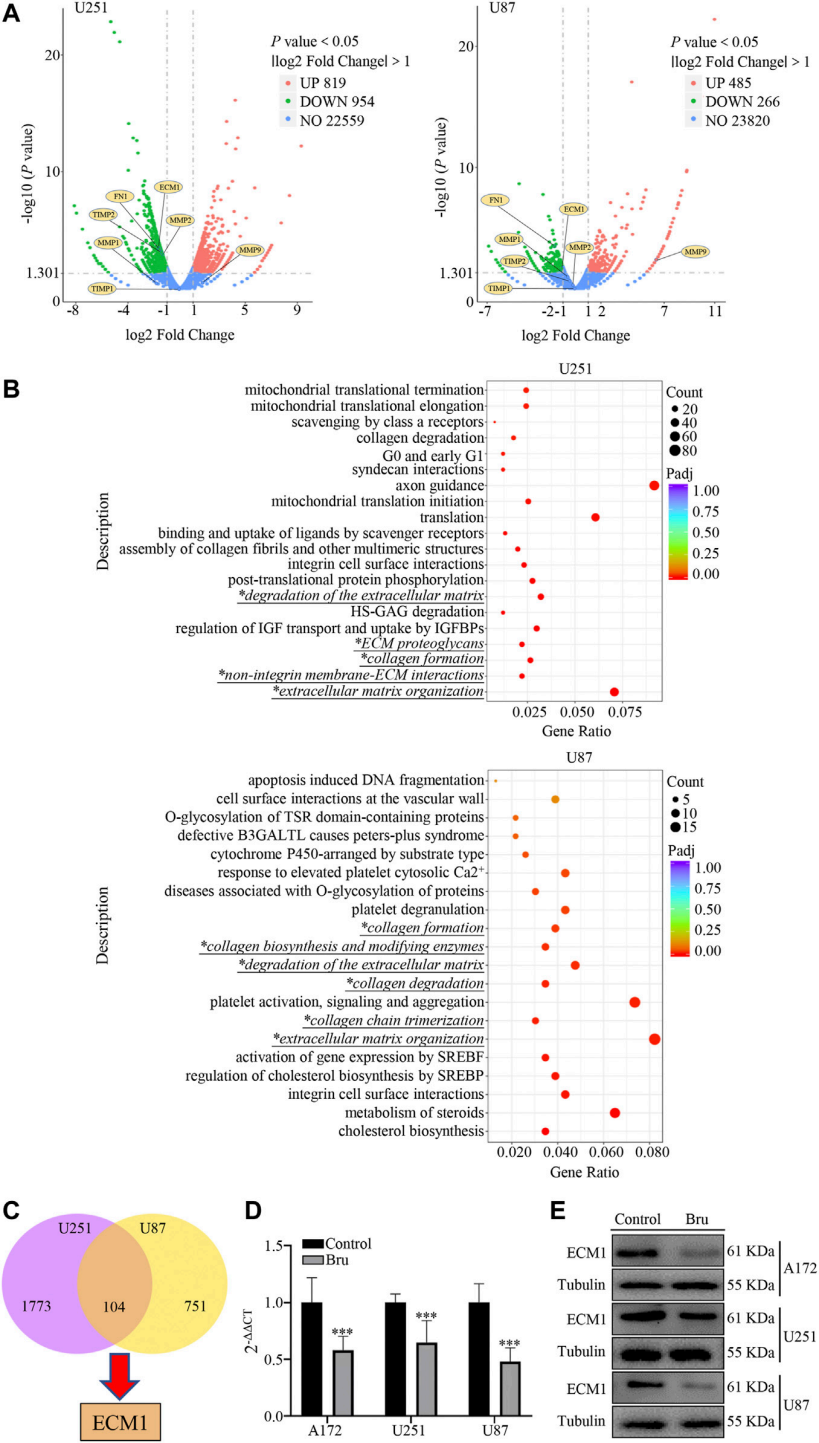
The expression of ECM1 was involved in Bru-treated GBM cells. **(A)** The volcano plots of RNAs in U251 and U87 cells. The Log2 absciss presented the differences existing in Control and Bru-treated group. The vertical axis showed the *p* value, which represented the significance of the difference. The expression of ECM1 and other related genes were shown as volcano plots to indicate the location of these genes. **(B)** Reactome enrichment analysis of the gene expression profiles in U251 and U87 cells. 20 pathways were shown to be significantly regulated in Control and Bru-treated group. Italic Script with * represented collagen and extracellular matrix related pathways. **(C)** Venn diagram analysis of the gene expression profiles in U251 and U87 cells. There were 104 genes co-expressed in these two GBM cell lines. **(D, E)** The cells were treated with Bru for 48 h and then subjected to qRT-PCR analysis and western blot assay to verify the expression of ECM1. *p < 0.05, **p < 0.01, ***p < 0.001 vs., Control group.

In order to further study the underlying mechanism of Bru’s action on GBM, we performed RNA sequencing array on GBM cells treated by Bru with IC_50_ for 48 h. A log2 fold change >1.0 and a *p* < 0.05 were used as cutoff criteria. The volcano plots results showed that Bru significantly changed the mRNA expression pattern in U251 (819 upregulated genes and 954 downregulated genes) and U87 (485 upregulated genes and 266 downregulated genes) cells (Figure 3A). Then, we subjected these differentially expressed genes to the Reactome pathway database (https://reactome.org), which provides molecular details of signal transduction, transport, DNA replication, metabolism, and other cellular processes as an ordered network of molecular transformations in a single, consistent data model. Reactome enrichment analysis indicated that the collagen and extracellular matrix related pathways (Italic Script with *) were mainly involved in Bru-treated GBM cells (Figure 3B). Subsequently, we used the Venn diagram analysis (VDA) to display the shared genes between U251 and U87 cells (Figure 3C, listed in Supplementary Table S1) and finally determined that ECM1, an important factor in extracellular matrix formation, changed obviously by Bru treatment. In order to verify the accuracy and repetition of RNA sequencing array results, three GBM cell lines treated with Bru for 48 h were subjected to perform qRT-PCR analysis and western blot assay. The results showed that, compared with Control group, Bru treatment significantly reduced the mRNA expression (Figure 3D) and protein level (Figure 3E) of ECM1 in these three GBM cell lines.

The results above showed that the expression of ECM1 had a close relationship with Bru treatment.

### ECM1 Silencing Suppressed Proliferation, Migration and Invasion of GBM Cells

**FIGURE 4:**
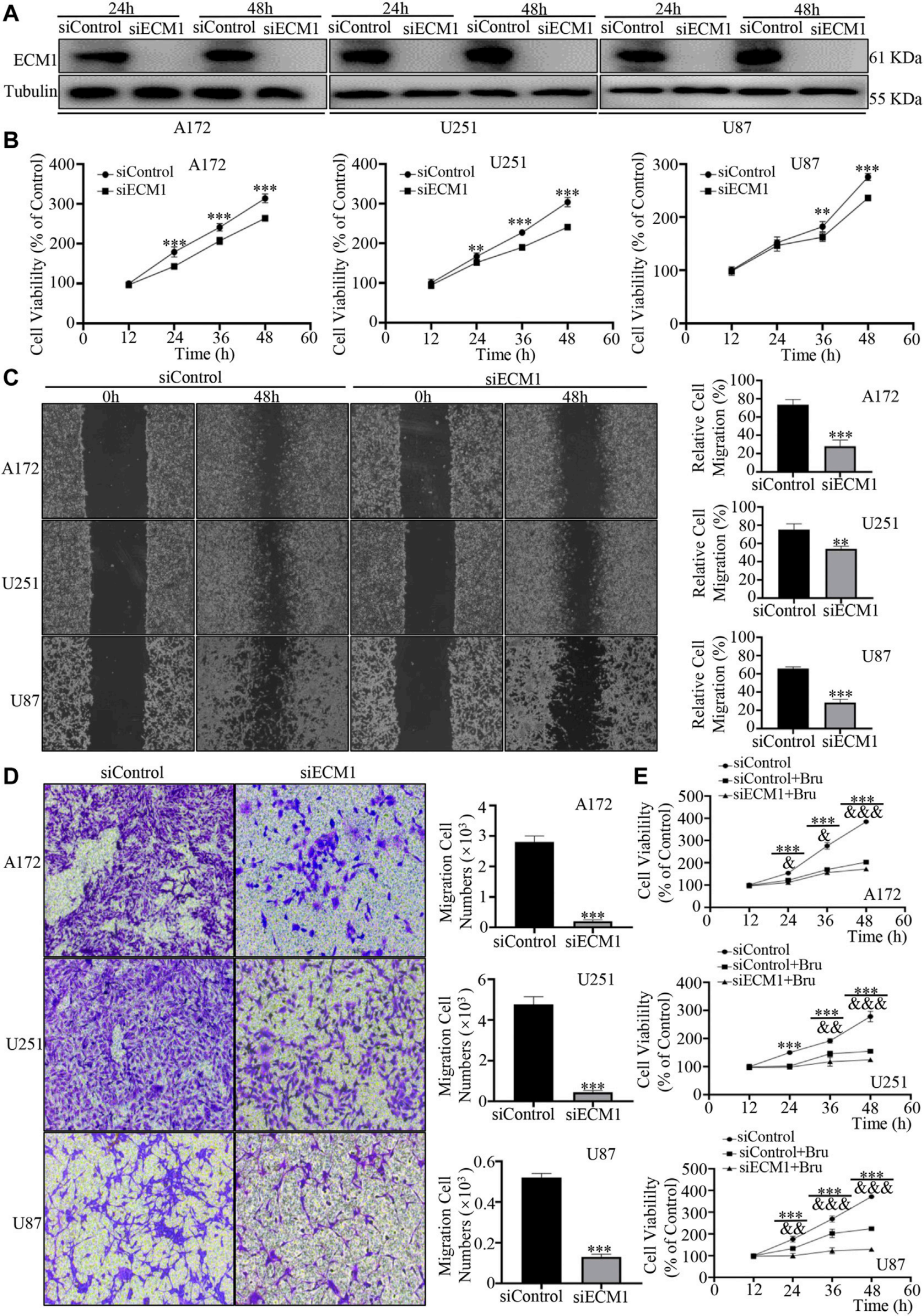
ECM1 silencing inhibited proliferation, migration and invasion of GBM cells. **(A)** The effect of ECM1 knockdown *via* siRNA silencing. Western blot analysis was used to measure the expression level of ECM1 in GBM cell lines transfected with siRNA for 24–48 h. **(B)** ECM1 knockdown *via* siRNA silencing inhibited the proliferation of A172 **(left)**, U251 **(middle)**, and U87 **(right)** cells. The cells were transfected with siControl or siECM1 for 12–60 h and then subjected to cell viability assay by CCK-8. **(C)** ECM1 knockdown suppressed the migration abilities of GBM cells. As described, the cells were subjected to cell migration assay by wound healing assay. The representative images for GBM cells were shown in Panel **(C)** **(left)**, and data from three independent experiments were showed in the histogram **(right)**. **(D)** ECM1 knockdown suppressed the invasive abilities of GBM cells. The cells were transfected with siControl or siECM1 for 48 h and then subjected to cell invasion assay. The representative images for GBM cell lines were shown in Panel **(D)** **(left)**, and data from three independent experiments were shown in the histogram **(right)**. **(E)** GBM cell lines were transfected with siECM1 for 24h, subsequently treated with Bru for 48–96h, and then subjected to cell proliferation assay by CCK-8. *p < 0.05, **p < 0.01, ***p < 0.001 vs., siControl group. ^&^ *p* < 0.05, ^&&^ *p* < 0.01, ^&&&^ *p* < 0.001 vs., siControl + Bru group.

To verify the effects of ECM1 on proliferation, migration, and invasion of GBM cells, ECM1 siRNA was transfected into the three GBM cell lines to down-regulate the expression of ECM1. Western blot analysis showed that the expression of ECM1 in siECM1 group was significantly lower than that in siControl group (Figure 4A). In cell viability assay, the three GBM cell lines showed significant reduction of cell viability after silencing ECM1 (Figure 4B). 48 h after siRNA transfection, the cell viability in siECM1 group was significantly reduced by 50.13 ± 4.47% in A172 cells (*p* < 0.001), 63.14 ± 6.20% in U251 cells (*p* < 0.001), and 39.45 ± 4.36% in U87 cells (*p* < 0.001), compared with siControl group. In order to explore whether ECM1 knockdown could inhibit the migration and invasion, as a novel cellular response to regulate cell proliferation, we knocked down the expression of ECM1 in GBM cell lines and subjected these cells to wound healing and transwell assays. The results showed that, compared with siControl group, the wound healing migration ability of GBM cells in siECM1 group was significantly reduced by 45.51 ± 6.03% in A172 cells (*p* < 0.001), 21.03 ± 2.55% in U251 cells (*p* < 0.01), and 37.35 ± 3.24% in U87 cells (*p* < 0.001) at 48 h (Figure 4C). In transwell assay, ECM1 silencing could significantly decrease cellular invasion ability of A172 cells by 92.75 ± 1.41% (*p* < 0.001), U251 cells by 90.68 ± 1.41% (*p* < 0.001), and U87 cells by 75.02 ± 1.09% (*p* < 0.001) at 48 h (Figure 4D). Moreover, we also found that ECM1 silencing was sufficient to increase the chemosensitivity of Bru by 31.12 ± 3.74% (*p* < 0.001), 30.10 ± 2.59% (*p* < 0.001), and 94.92 ± 4.50% (*p* < 0.001) at 48 h in A172, U251, and U87, respectively (Figure 4E).

These findings suggested that ECM1 played an important role in promoting the growth and invasion of GBM cells, and ECM1 silencing could sensitize GBM cells to Bru treatment.

### Up-Regulation of ECM1 Promoted Proliferation, Migration and Invasion of GBM Cells

**FIGURE 5:**
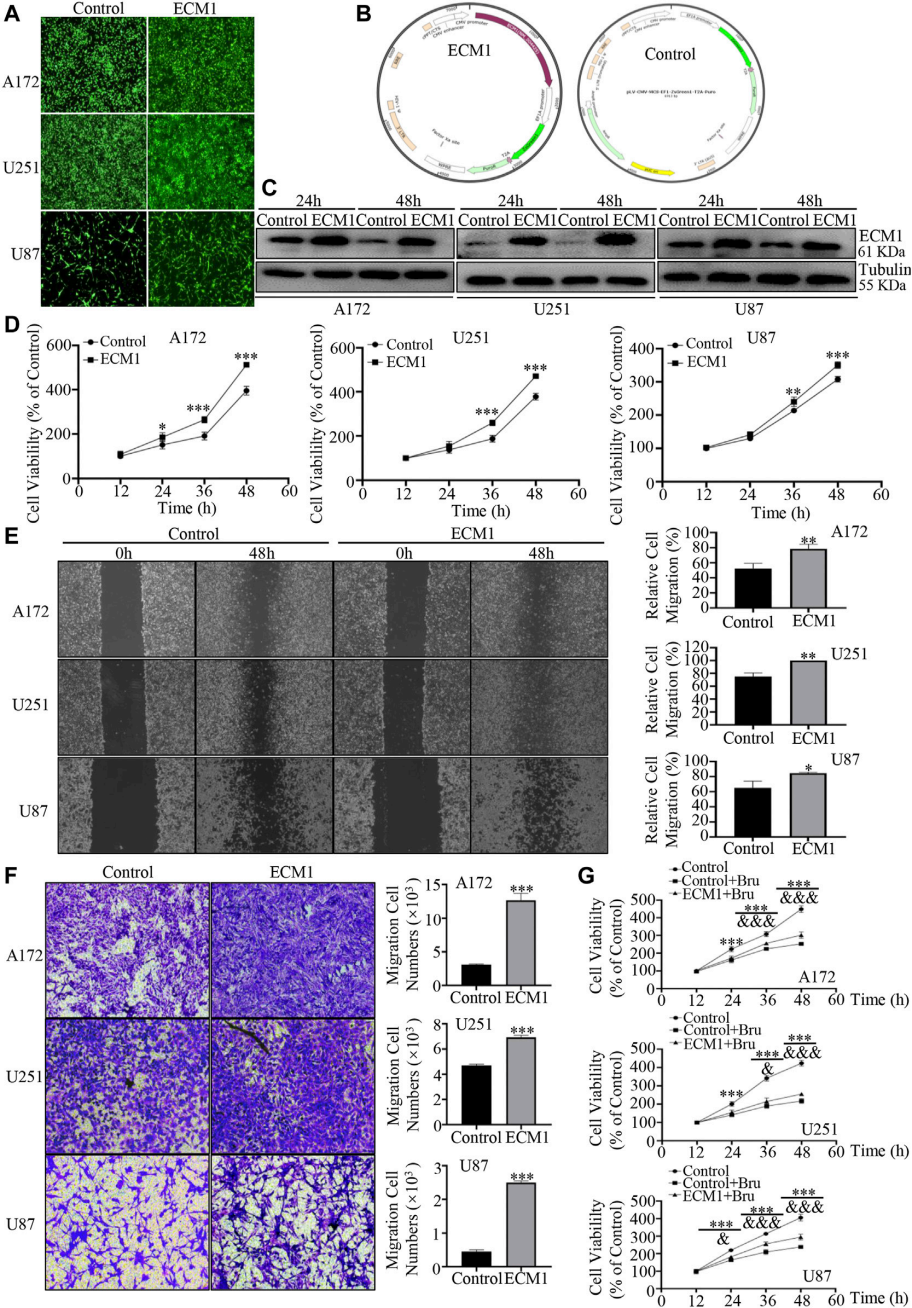
Overexpression of ECM1 promoted proliferation, migration and invasion of GBM cells. **(A)** After transfecting with lentivirus for 96h, the transfection efficiency of lentivirus in GBM cells was observed. **(B)** The structures of the plasmid overexpressing ECM1 (pLV-CMV-ECM1-EF1-ZsGreen1-T2A-Puro) and the Control plasmid (pLV-CMV-MCS-EF1-ZsGreen1-T2A-Puro) were shown in Panel **(B)**. **(C)** Western blot analysis was used to detect the expression level of ECM1 in GBM cells transfected with pLV-vector (Control) or pLV-ECM1 (ECM1). **(D)** Overexpression of ECM1 promoted the proliferation of A172 **(left)**, U251 **(middle)**, and U87 **(right)** cells. The cells were transfected with pLV-vector (Control) or pLV-ECM1 (ECM1) for 12–60h, and cell viability was determined by CCK-8. **(E)** Overexpression of ECM1 promoted the migration abilities of GBM cells. The wound-healing assay was used to detect the migration abilities of GBM cells. The images of GBM cell migration were shown in Panel **(E)** **(left)**, and data from three independent experiments were shown in the histogram **(right)**. **(F)** Overexpression of ECM1 was sufficient for the invasive abilities of GBM cells. The cells were transfected with lentivirus for 48 h and subjected to cell invasion assay. The images of GBM cell invasion were shown in Panel **(F)** **(left)**, and data from three independent experiments were shown in the histogram **(right)**. **(G)** GBM cell lines were infected with pLV-ECM1 for 24 h, treated with Bru for 48–96 h, and then subjected to cell proliferation assay by CCK-8. *p < 0.05, **p < 0.01, ***p < 0.001 vs., Control group. ^&^ *p* < 0.05, ^&&^ *p* < 0.01, ^&&&^ *p* < 0.001 vs, Control + Bru group.

In order to further investigate the protein expression profile of ECM1 on GBM cells, the three GBM cell lines (A172, U251, U87) were transfected with lentivirus to overexpress ECM1. After transfection for 96h, most of the cells showed positive signals of ZsGreen1, indicating that the lentiviruses infected GBM cells efficiently (Figure 5A). The structures of the plasmid overexpressing ECM1 (pLV-CMV-ECM1-EF1-ZsGreen1-T2A-Puro) and the Control plasmid (pLV-CMV-MCS-EF1-ZsGreen1-T2A-Puro) were shown in Figure 5B. Western blot analysis was also used to measure the transfection efficiency, and the results showed that the expression level of ECM1 was significantly up-regulated in ECM1 group (Figure 5C). In cell viability assay, the results showed that overexpressed ECM1 could distinctively promote cell proliferation, compared with Control group (Figure 5D). Cell viability of GBM cells in ECM1 group was significantly increased by 117.28 ± 6.40% in A172 cells (*p* < 0.001), 92.73 ± 8.70% in U251 cells (*p* < 0.001), and 42.53 ± 5.74% in U87 cells (*p* < 0.001) at 48 h, compared with Control group. In addition, the wound healing assay showed that overexpression of ECM1 enhanced the migration ability of GBM cells by 26.14 ± 5.63% in A172 cells (*p* < 0.01), 24.85 ± 3.24% in U251 cells (*p* < 0.01), and 19.38 ± 4.80% in U87 cells (*p* < 0.05) at 48 h (Figure 5E). As shown in Figure 5F, ECM1 overexpression could significantly increase cellular invasion ability of A172 cells by 308.73 ± 19.17% (*p* < 0.001), U251 cells by 47.53 ± 1.55% (*p* < 0.001), and U87 cells by 459.72 ± 32.66% (*p* < 0.001) at 48 h. Besides, we also found that up-regulating the expression of ECM1 was sufficient to reduce chemosensitivity of Bru by 49.78 ± 9.23% (*p* < 0.001), 37.78 ± 7.15% (*p* < 0.001), and 56.21 ± 9.43% (*p* < 0.001) at 48 h in A172, U251, and U87, respectively (Figure 5G).

All these results above revealed that ECM1 was required for the growth of GBM cells and overexpressed ECM1 could reverse the inhibitory effect of Bru treatment on GBM cells.

### Effect of Bru Treatment on Migration-Related Biomarkers

**FIGURE 6:**
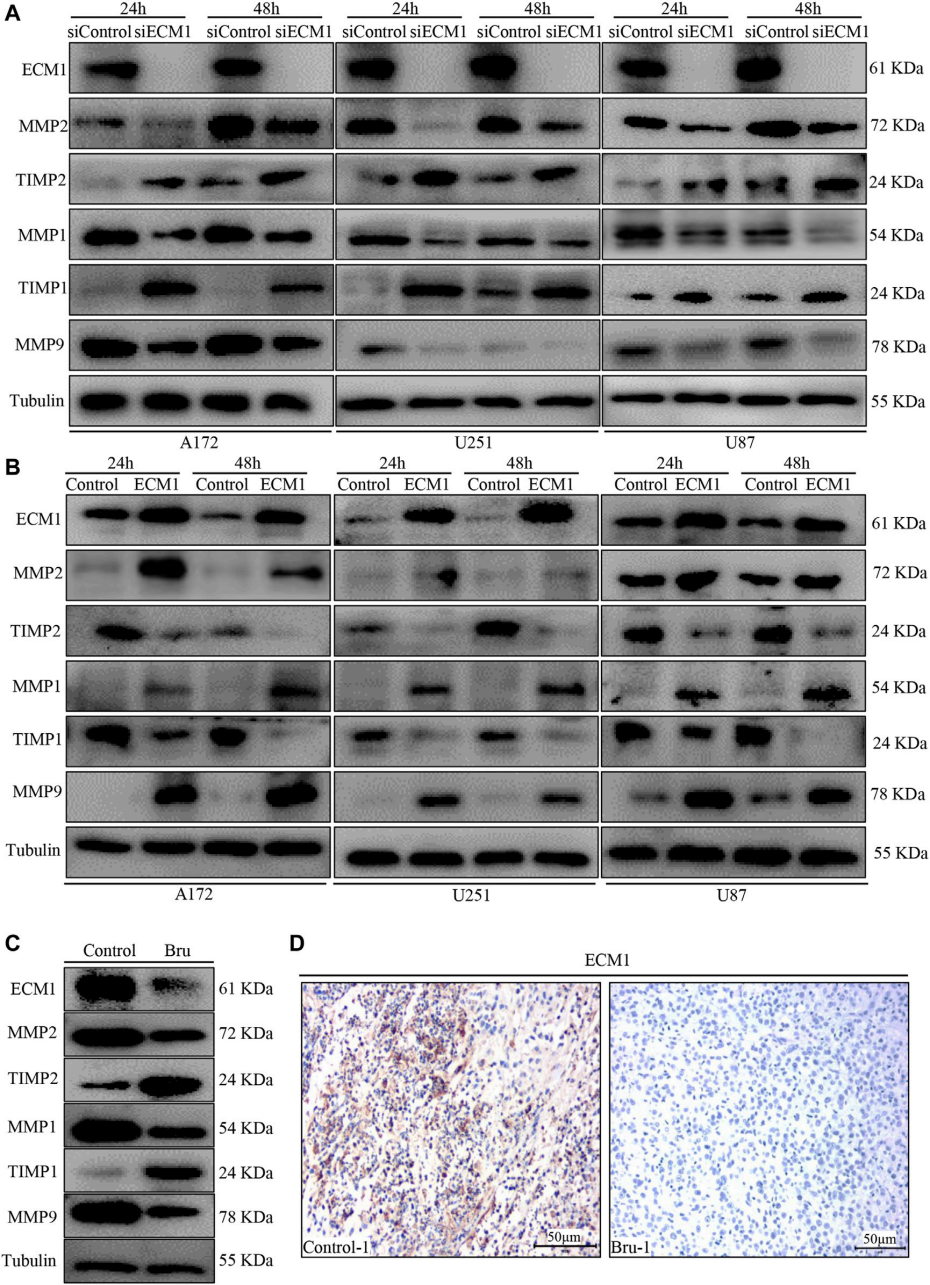
The effect of ECM1 on invasion-related biomarkers. **(A)** Cells were transfected with siControl or siECM1 for 24–48 h and subjected to immunoblot analysis for measuring the expression of indicated proteins. **(B)** Cells were infected with pLV-vector (Control) or pLV-ECM1 (ECM1) for 24–48 h and then subjected to immunoblot analysis for measuring the expression of indicated proteins. **(C)** Immunoblot analyses showing ECM1, MMP2, TIMP2, MMP1, TIMP1 and MMP9 expression levels in xenograft tumors. The blots shown here were representatives of the average of five subcutaneously transplanted tumors in nude mice. **(D)** Representative images of IHC staining showed that Bru decreased the levels of ECM1 in subcutaneously transplanted xenograft tumors ().

In order to further explore the molecular mechanism of Bru treatment, we performed western blot to examine the expression of migration-related biomarkers, including MMP1, MMP2, MMP9, TIMP1, and TIMP2. The results showed that knockdown of ECM1 significantly reduced the expression of MMP1, MMP2 and MMP9, while significantly increasing the expression of TIMP1 and TIMP2 (Figure 6A). On the contrary, overexpression of ECM1 significantly increased the protein levels of MMP1, MMP2 and MMP9, while reducing the expression of TIMP1 and TIMP2 (Figure 6B). The expression of ECM1 and other related genes in RNA sequencing array were also shown in Figure 3A (indicated as volcano plots). In addition, as shown in Figure 6C, the expression levels of ECM1, MMP1, MMP2 and MMP9 in xenograft tumors tissues from Bru-treated group significantly decreased, compared with the Control group, while the expression levels of TIMP1 and TIMP2 significantly increased (Figure 6C). Consistently, IHC staining also showed that the expression levels of ECM1 in xenograft tumors from Bru-treated mice decreased, compared with the levels in tumors from Control group (n = 5, Figure 6D and Supplementary Figure S1A).

These results demonstrated that Bru could exert its inhibitory effect by down-regulating the expression of ECM1 *in vitro* and *in vivo*.

### The Cytotoxic Effect of Bru Treatment on Human Primary GBM Cells was Related With the Expression of ECM1

**FIGURE 7:**
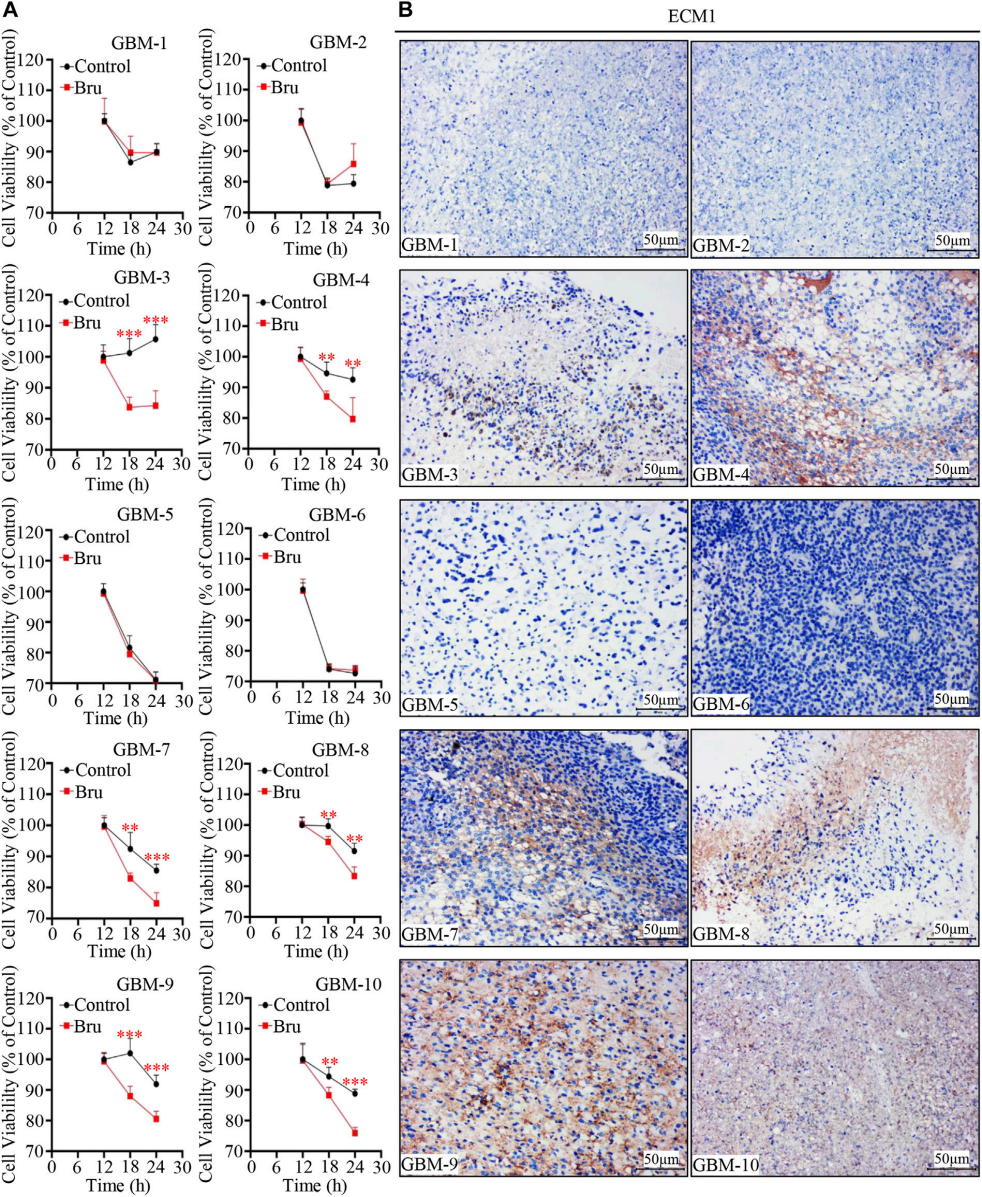
Cytotoxic effect of Bru on human primary GBM cells. **(A)** 10 human primary GBM cell samples were treated with 50 nM Bru. Cell viability was measured at the indicated time points by CCK-8 assay. Bru decreased viable cells significantly in six of 10 human primary GBM cell samples. **(B)** Representative images of IHC staining showed that ECM1 expression was high in the majority of Bru-treated sensitive human primary GBM cell samples. *p < 0.05, **p < 0.01, ***p < 0.001 vs, Control group.

In order to determine whether the expression of ECM1 was an important prognostic factor for Bru treatment on human primary GBM cells, we collected human GBM tissue samples to culture *in vitro*, and the cells were then treated with Bru for 12–24 h and subjected to cell viability assay. The results showed that Bru treatment (50 nM) resulted in a viability decrease in six out of 10 human GBM tissue samples (Figure 7A). The HE staining results were shown in Supplementary Figure S1B, and the expression of ECM1 protein was high in the majority of these Bru-treated sensitive human primary GBM cells (Figure 7B). Interestingly, based on the clinical characteristics of GBM patients, tumor cells collected from the MGMT-negative patients were also sensitive to Bru treatment at 48h, indicating that Bru successfully inhibited the proliferation of TMZ-resistant human primary GBM cells (case No. 4, 8, 10 in Table 1).

In all, these results suggested that Bru was also effective in the treatment of human primary GBM cells.

## Discussion

In recent years, traditional Chinese herbal medicines have been found to hold the characteristics of low toxicity and few side effects, which gradually make them the research hotspot in the field of anticancer treatment. Bru, as one of the Chinese herbal extracts, has been shown to possess significant therapeutic effects in the treatment of multiple cancer diseases (Ren et al., 2011; Evans et al., 2018). Our study demonstrated that Bru exerted a significant inhibitory effect on the growth of GBM cell lines *in vitro* and *in vivo*, as well as on human primary GBM cells, indicating that Bru has potential clinical application as a novel drug for the treatment of glioblastomas.

Bru is a biologically active triterpene that exhibits significant pharmacological effects in anti-tumor, anti-malarial, anti-inflammatory, anti-viral, and insecticidal applications (Tang et al., 2014). In several studies, Bru was considered as an unparalleled inhibitor of the Nrf2 pathway, exhibiting growth inhibition and pro-apoptotic effects in a variety of cancer cells (Evans et al., 2018; Cai et al., 2019). By promoting ROS production and down-regulating Nrf2 expression, Bru significantly inhibited the growth of lung cancer and pituitary tumor cells *in vitro* and *in vivo* (Sun et al., 2016; Wu et al., 2021). Similar results were also shown in the field of glioma treatment (Tang et al., 2020). Despite several studies have shown that anti-tumor capacity of Bru were mainly mediated by down-regulating the expression of Nrf2, reports about majority of Nrf2 inhibitors were shown to be less promising in clinical trials (Preul et al., 1993), suggesting that Bru treatment may be associated with a more complex mechanism of signaling that need to explored. Besides, the mechanism of drug action in anti-cancers is diverse, and the detailed pharmacological effects of Bru cannot be fully explained by the studies conducted only on one target of action.

In our research, the mechanism of Bru in the treatment of GBM was verified by RNA-sequence array and the results showed that Bru could induce cell apoptosis of GBM *via* downregulating the expression of ECM1, a 85 kDa secreted glycoprotein first isolated from the mouse osteoblast cell line MN7 in 1994 (Mathieu et al., 1994). ECM1 plays an important role in the regulation of extracellular matrix formation, cell adhesion, cell signal transmission, and tissue differentiation and maturation (Sercu et al., 2008b). A number of current studies have shown that ECM1 could promote tumor cell proliferation and invasion in malignant tumors, including digestive system (Wang et al., 2003; Chen et al., 2011; Xiong et al., 2012), respiratory system (Wang et al., 2003), breast cancer, and melanoma (Lal et al., 2009; Lal et al., 2013; Steinhaeuser et al., 2020). On the contrary, down-regulating the expression of ECM1 could significantly suppress the proliferation, migration, and invasion of tumor cells (Huang et al., 2019; Yang et al., 2020), and the underlying mechanism could be mainly related to MMP signaling pathway (Sercu et al., 2008a; Abdel Salam et al., 2015). Our study results also proved that down-regulating the expression of ECM1 could suppress GBM cell proliferation, migration, and invasion, sensitizing the cells to Bru treatment as well. Overexpressing ECM1 could reverse this effect and decrease the efficacy of Bru treatment. Moreover, Bru-induced cell death was also effective on human primary GBM cells, including those collected from MGMT-negative patients. These results above suggested that Bru may offer a new supplementary approach for treating TMZ-resistant glioblastomas.

On the other hand, some research also indicated that Bru could act as an inflammation inhibitor, which protected pancreatic β-cells against cytokines-induced (IL-1β and IFN-γ) oxidative stress and iNOS expression *in vitro* (Turpaev and Welsh, 2015). In addition, the study by Liang et al. showed that Bru possessed its protective role in a rat model of oxygen-induced retinopathy of prematurity *via* counteracting microglial activation and cellular angiogenesis (Liang and Wang, 2021). Inversely, a study showed that Bru could exert its inhibitory effect in breast cancer by stimulating lymphocyte migration towards tumor tissues and activating anti-tumor immune responses *in vivo* (Bovilla et al., 2021). Obviously, this discrepancy can’t be briefly explained by Bru-dependent inhibition of Nrf2 and whether the inflammation-related effects also play important role in Bru treatment still need further study. An earlier study indicated that inflammation was also involved in accelerating cancer progression in GBM (Ham et al., 2019). Whether Bru exerts its pharmacological effects by regulating other cellular components within the GBM microenvironment, such as immune cells or vascular endothelial cells, was not investigated in the current study. This is a limitation of our study and an important area for further investigation.

The most challenging bottleneck in the treatment of central nervous system tumors is the ability of drugs to penetrate the blood-brain barrier (BBB). Although we established that Bru had a significant inhibitory effect on the proliferation, growth and invasion of GBM cells, other studies have found that Bru is not able to pass through the BBB. This greatly limits the application of Bru for *in vivo* treatment of glioblastomas (Guo et al., 2017). Novel drug delivery carriers, such as exosomes, the nano-sized membrane particles secreted by cells, do however possess the ability to cross the BBB with low cytotoxicity (Nam et al., 2020; Peng et al., 2020). These carriers may provide new solutions to this major limitation for the clinical application of Bru and can be investigated in the future (Kim et al., 2016).

## Conclusion

In summary, this study established the anticancer effects of Bru on GBM cells by down-regulating the expression of ECM1. The findings provide us with novel insights into the mechanism of Bru function, as well as with a potential therapeutic drug for the medical management of glioblastoma cells.

## Data Availability

The datasets presented in this study can be found in online repositories. The names of the repository/repositories and accession number(s) can be found below: https://www.ncbi.nlm.nih.gov/geo/query/acc.cgi?acc=GSE184859.

## Supplementary Material

Supplemental Figure S1
**(A)** Representative images of IHC staining showed that Bru decreased the levels of ECM1 in subcutaneously transplanted xenograft tumors. **(B)** Representative images of HE staining of 10 human primary GBM cell samples

## References

[B1] Abdel Salam R. El-Badry N. Rizk A. El-Sedfy A. Kamel N. El-Abd E. (2015). Expression of ECM1 and MMP-2 in Follicular Thyroid Lesions Among Egyptians. Cancer Biomark 15, 441–458. 10.3233/cbm-150481 25812648 PMC12965080

[B2] Bovilla V. R. Kuruburu M. G. Bettada V. G. Krishnamurthy J. Sukocheva O. A. Thimmulappa R. K. (2021). Targeted Inhibition of Anti-inflammatory Regulator Nrf2 Results in Breast Cancer Retardation *In Vitro* and *In Vivo* . Biomedicines 9, 1119. 10.3390/biomedicines9091119 34572304 PMC8471069

[B3] Cai S. J. Liu Y. Han S. Yang C. (2019). Brusatol, an NRF2 Inhibitor for Future Cancer Therapeutic. Cell Biosci. 9, 45. 10.1186/s13578-019-0309-8 31183074 PMC6554866

[B4] Chen H. Jia W.-D. Li J.-S. Wang W. Xu G.-L. Ma J.-L. (2011). Extracellular Matrix Protein 1, a Novel Prognostic Factor, Is Associated with Metastatic Potential of Hepatocellular Carcinoma. Med. Oncol. 28, 318–325. 10.1007/s12032-010-9763-1 21128013

[B5] Chen H. M. Lai Z. Q. Liao H. J. Xie J. H. Xian Y. F. Chen Y. L. (2018). Synergistic Antitumor Effect of Brusatol Combined with Cisplatin on Colorectal Cancer Cells. Int. J. Mol. Med. 41, 1447–1454. 10.3892/ijmm.2018.3372 29328398 PMC5819912

[B6] Evans J. P. Winiarski B. K. Sutton P. A. Jones R. P. Ressel L. Duckworth C. A. (2018). The Nrf2 Inhibitor Brusatol Is a Potent Antitumour Agent in an Orthotopic Mouse Model of Colorectal Cancer. Oncotarget 9, 27104–27116. 10.18632/oncotarget.25497 29930754 PMC6007465

[B7] Gilbert M. R. Wang M. Aldape K. D. Stupp R. Hegi M. E. Jaeckle K. A. (2013). Dose-dense Temozolomide for Newly Diagnosed Glioblastoma: a Randomized Phase III Clinical Trial. J. Clin. Oncol. 31, 4085–4091. 10.1200/jco.2013.49.6968 24101040 PMC3816958

[B8] Guo N. Zhang X. Bu F. Wang L. Cao Z. Geng C. (2017). Determination of Brusatol in Plasma and Tissues by LC-MS Method and its Application to a Pharmacokinetic and Distribution Study in Mice. J. Chromatogr. B Analyt Technol. Biomed. Life Sci. 1053, 20–26. 10.1016/j.jchromb.2017.04.012 28407533

[B9] Hall I. H. Lee K. H. Eigebaly S. A. Imakura Y. Sumida Y. Wu R. Y. (1979). Antitumor Agents. XXXIV: Mechanism of Action of Bruceoside A and Brusatol on Nucleic Acid Metabolism of P-388 Lymphocytic Leukemia Cells. J. Pharm. Sci. 68, 883–887. 10.1002/jps.2600680726 458610

[B10] Hall I. H. Lee K. H. Okano M. Sims D. Ibuka T. Liou Y. F. (1981). Antitumor Agents XLII: Comparison of Antileukemic Activity of Helenalin, Brusatol, and Bruceantin and Their Esters on Different Strains of P-388 Lymphocytic Leukemic Cells. J. Pharm. Sci. 70, 1147–1150. 10.1002/jps.2600701014 7299649

[B11] Ham S. W. Jeon H. Y. Jin X. Kim E. J. Kim J. K. Shin Y. J. (2019). TP53 Gain-Of-Function Mutation Promotes Inflammation in Glioblastoma. Cell Death Differ 26, 409–425. 10.1038/s41418-018-0126-3 29786075 PMC6370770

[B12] Hegi M. E. Diserens A. C. Gorlia T. Hamou M. F. De Tribolet N. Weller M. (2005). MGMT Gene Silencing and Benefit from Temozolomide in Glioblastoma. N. Engl. J. Med. 352, 997–1003. 10.1056/NEJMoa043331 15758010

[B13] Hegi M. E. Genbrugge E. Gorlia T. Stupp R. Gilbert M. R. Chinot O. L. (2019). MGMT Promoter Methylation Cutoff with Safety Margin for Selecting Glioblastoma Patients into Trials Omitting Temozolomide: A Pooled Analysis of Four Clinical Trials. Clin. Cancer Res. 25, 1809–1816. 10.1158/1078-0432.Ccr-18-3181 30514777 PMC8127866

[B14] Huang W. Huang Y. Gu J. Zhang J. Yang J. Liu S. (2019). miR-23a-5p Inhibits Cell Proliferation and Invasion in Pancreatic Ductal Adenocarcinoma by Suppressing ECM1 Expression. Am. J. Transl Res. 11, 2983–2994. 31217868 PMC6556669

[B15] Kaur E. Nair J. Ghorai A. Mishra S. V. Achareker A. Ketkar M. (2020). Inhibition of SETMAR-H3K36me2-NHEJ Repair axis in Residual Disease Cells Prevent Glioblastoma Recurrence. Neuro Oncol. 22, 1785–1796. 10.1093/neuonc/noaa128 32458986 PMC7746947

[B16] Keles G. E. Berger M. S. (2004). Advances in Neurosurgical Technique in the Current Management of Brain Tumors. Semin. Oncol. 31, 659–665. 10.1053/j.seminoncol.2004.07.008 15497119

[B17] Kim M. S. Haney M. J. Zhao Y. Mahajan V. Deygen I. Klyachko N. L. (2016). Development of Exosome-Encapsulated Paclitaxel to Overcome MDR in Cancer Cells. Nanomedicine 12, 655–664. 10.1016/j.nano.2015.10.012 26586551 PMC4809755

[B18] Lal G. Contreras P. G. Kulak M. Woodfield G. Bair T. Domann F. E. (2013). Human Melanoma Cells Over-express Extracellular Matrix 1 (ECM1) Which Is Regulated by TFAP2C. PloS one 8, e73953. 10.1371/journal.pone.0073953 24023917 PMC3759440

[B19] Lal G. Hashimi S. Smith B. J. Lynch C. F. Zhang L. Robinson R. A. (2009). Extracellular Matrix 1 (ECM1) Expression Is a Novel Prognostic Marker for Poor Long-Term Survival in Breast Cancer: a Hospital-Based Cohort Study in Iowa. Ann. Surg. Oncol. 16, 2280–2287. 10.1245/s10434-009-0533-2 19521735

[B20] Liang X. Wang R. (2021). The Nrf2 Inhibitor Brusatol Has a Protective Role in a Rat Model of Oxygen-Induced Retinopathy of Prematurity. Vis. Neurosci. 38, E002. 10.1017/s095252382100002x 33729121

[B21] Liu J. Eckert M. A. Harada B. T. Liu S. M. Lu Z. Yu K. (2018). m6A mRNA Methylation Regulates AKT Activity to Promote the Proliferation and Tumorigenicity of Endometrial Cancer. Nat. Cell Biol. 20, 1074–1083. 10.1038/s41556-018-0174-4 30154548 PMC6245953

[B22] Lu Z. Lai Z. Q. Leung A. W. N. Leung P. S. Li Z. S. Lin Z. X. (2017). Exploring Brusatol as a New Anti-pancreatic Cancer Adjuvant: Biological Evaluation and Mechanistic Studies. Oncotarget 8, 84974–84985. 10.18632/oncotarget.17761 29156697 PMC5689587

[B23] Mata-Greenwood E. Cuendet M. Sher D. Gustin D. Stock W. Pezzuto J. M. (2002). Brusatol-mediated Induction of Leukemic Cell Differentiation and G(1) Arrest Is Associated with Down-Regulation of C-Myc. Leukemia 16, 2275–2284. 10.1038/sj.leu.2402696 12399973

[B24] Mathieu E. Meheus L. Raymackers J. Merregaert J. (1994). Characterization of the Osteogenic Stromal Cell Line MN7: Identification of Secreted MN7 Proteins Using Two-Dimensional Polyacrylamide Gel Electrophoresis, Western Blotting, and Microsequencing. J. Bone Miner Res. 9, 903–913. 10.1002/jbmr.5650090616 8079665

[B25] Mcaleenan A. Kelly C. Spiga F. Kernohan A. Cheng H. Y. Dawson S. (2021). Prognostic Value of Test(s) for O6-Methylguanine-DNA Methyltransferase (MGMT) Promoter Methylation for Predicting Overall Survival in People with Glioblastoma Treated with Temozolomide. Cochrane Database Syst. Rev. 3, CD013316. 10.1002/14651858.CD013316.pub2 33710615 PMC8078495

[B26] Nam G. H. Choi Y. Kim G. B. Kim S. Kim S. A. Kim I. S. (2020). Emerging Prospects of Exosomes for Cancer Treatment: From Conventional Therapy to Immunotherapy. Adv. Mater. 32, e2002440. 10.1002/adma.202002440 33015883

[B27] Olayanju A. Copple I. M. Bryan H. K. Edge G. T. Sison R. L. Wong M. W. (2015). Brusatol Provokes a Rapid and Transient Inhibition of Nrf2 Signaling and Sensitizes Mammalian Cells to Chemical Toxicity-Implications for Therapeutic Targeting of Nrf2. Free Radic. Biol. Med. 78, 202–212. 10.1016/j.freeradbiomed.2014.11.003 25445704 PMC4291150

[B28] Peng H. Ji W. Zhao R. Yang J. Lu Z. Li Y. (2020). Exosome: a Significant Nano-Scale Drug Delivery Carrier. J. Mater. Chem. B 8, 7591–7608. 10.1039/d0tb01499k 32697267

[B29] Preul M. C. Stratford J. Bertrand G. Feindel W. (1993). Neurosurgeon as Innovator: William V. Cone (1897-1959). J. Neurosurg. 79, 619–631. 10.3171/jns.1993.79.4.0619 8410237

[B30] Ren D. Villeneuve N. F. Jiang T. Wu T. Lau A. Toppin H. A. (2011). Brusatol Enhances the Efficacy of Chemotherapy by Inhibiting the Nrf2-Mediated Defense Mechanism. Proc. Natl. Acad. Sci. U S A. 108, 1433–1438. 10.1073/pnas.1014275108 21205897 PMC3029730

[B31] Sercu S. Zhang L. Merregaert J. (2008a). The Extracellular Matrix Protein 1: its Molecular Interaction and Implication in Tumor Progression. Cancer Invest. 26, 375–384. 10.1080/07357900701788148 18443958

[B32] Sercu S. Zhang M. Oyama N. Hansen U. Ghalbzouri A. E. Jun G. (2008b). Interaction of Extracellular Matrix Protein 1 with Extracellular Matrix Components: ECM1 Is a Basement Membrane Protein of the Skin. J. Invest. Dermatol. 128, 1397–1408. 10.1038/sj.jid.5701231 18200062

[B33] Steinhaeuser S. S. Morera E. Budkova Z. Schepsky A. Wang Q. Rolfsson O. (2020). ECM1 Secreted by HER2-Overexpressing Breast Cancer Cells Promotes Formation of a Vascular Niche Accelerating Cancer Cell Migration and Invasion. Lab. Invest. 100, 928–944. 10.1038/s41374-020-0415-6 32203150

[B34] Sun X. Wang Q. Wang Y. Du L. Xu C. Liu Q. (2016). Brusatol Enhances the Radiosensitivity of A549 Cells by Promoting ROS Production and Enhancing DNA Damage. Int. J. Mol. Sci. 17, 997. 10.3390/ijms17070997 PMC4964373 27347930

[B35] Tang W. Xie J. Xu S. Lv H. Lin M. Yuan S. (2014). Novel Nitric Oxide-Releasing Derivatives of Brusatol as Anti-inflammatory Agents: Design, Synthesis, Biological Evaluation, and Nitric Oxide Release Studies. J. Med. Chem. 57, 7600–7612. 10.1021/jm5007534 25179783

[B36] Tang X. Fu X. Liu Y. Yu D. Cai S. J. Yang C. (2020). Blockade of Glutathione Metabolism in IDH1-Mutated Glioma. Mol. Cancer Ther. 19, 221–230. 10.1158/1535-7163.Mct-19-0103 31548295 PMC6946871

[B37] Turpaev K. Welsh N. (2015). Brusatol Inhibits the Response of Cultured Beta-Cells to Pro-inflammatory Cytokines *In Vitro* . Biochem. Biophys. Res. Commun. 460, 868–872. 10.1016/j.bbrc.2015.03.124 25824046

[B38] Wang L. Yu J. Ni J. Xu X. M. Wang J. Ning H. (2003). Extracellular Matrix Protein 1 (ECM1) Is Over-expressed in Malignant Epithelial Tumors. Cancer Lett. 200, 57–67. 10.1016/s0304-3835(03)00350-1 14550953

[B39] Wang M. Shi G. Bian C. Nisar M. F. Guo Y. Wu Y. (2018). UVA Irradiation Enhances Brusatol-Mediated Inhibition of Melanoma Growth by Downregulation of the Nrf2-Mediated Antioxidant Response. Oxid Med. Cell Longev 2018, 9742154. 10.1155/2018/9742154 29670684 PMC5835260

[B40] Wen P. Y. Kesari S. (2008). Malignant Gliomas in Adults. N. Engl. J. Med. 359, 492–507. 10.1056/NEJMra0708126 18669428

[B41] Wick A. Kessler T. Platten M. Meisner C. Bamberg M. Herrlinger U. (2020). Superiority of Temozolomide over Radiotherapy for Elderly Patients with RTK II Methylation Class, MGMT Promoter Methylated Malignant Astrocytoma. Neuro Oncol. 22, 1162–1172. 10.1093/neuonc/noaa033 32064499 PMC7594575

[B42] Wu Z. Xu Y. Xu J. Lu J. Cai L. Li Q. (2021). Brusatol Inhibits Tumor Growth and Increases the Efficacy of Cabergoline against Pituitary Adenomas. Oxid Med. Cell Longev 2021, 6696015. 10.1155/2021/6696015 34221237 PMC8221873

[B43] Xiong G. P. Zhang J. X. Gu S. P. Wu Y. B. Liu J. F. (2012). Overexpression of ECM1 Contributes to Migration and Invasion in Cholangiocarcinoma Cell. Neoplasma 59, 409–415. 10.4149/neo_2012_053 22489696

[B44] Yang H. Huang Y. He J. Chai G. Di Y. Wang A. (2020). MiR-486-3p Inhibits the Proliferation, Migration and Invasion of Retinoblastoma Cells by Targeting ECM1. Biosci. Rep. 40, BSR20200392. 10.1042/bsr20200392 32401301 PMC7273916

[B45] Ye R. Dai N. He Q. Guo P. Xiang Y. Zhang Q. (2018). Comprehensive Anti-tumor Effect of Brusatol through Inhibition of Cell Viability and Promotion of Apoptosis Caused by Autophagy via the PI3K/Akt/mTOR Pathway in Hepatocellular Carcinoma. Biomed. Pharmacother. 105, 962–973. 10.1016/j.biopha.2018.06.065 30021391

