# Supplementary figures and images for: Brusatol Inhibits Proliferation and Invasion of Glioblastoma by Down-Regulating the Expression of ECM1
Source: Front Pharmacol. 2021 Dec 14;12:775680. doi: 10.3389/fphar.2021.775680 (PMC8713816; doi:10.3389/fphar.2021.775680)

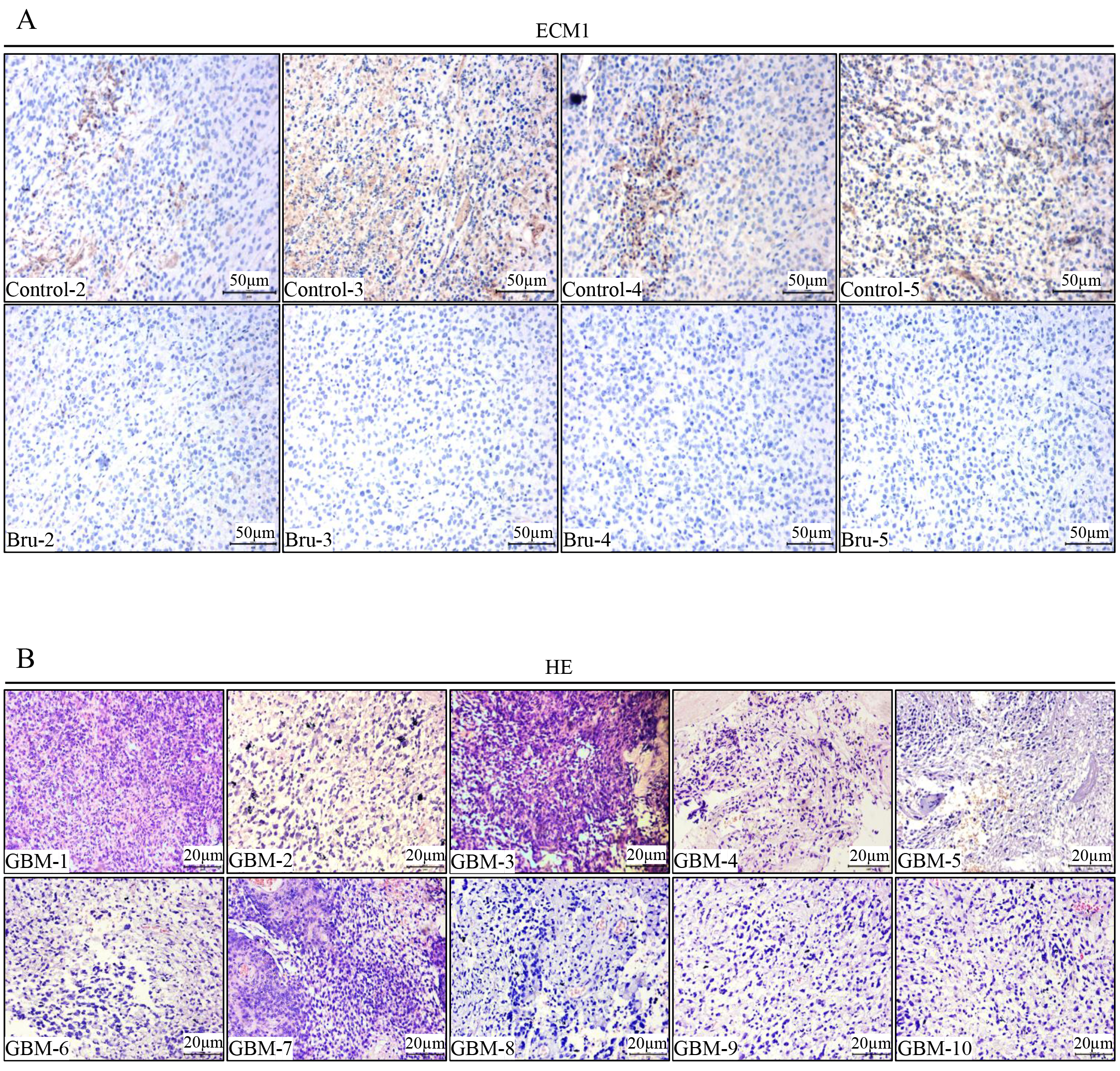

Supplement: Supplementary file 1 [file Image1.JPEG]
